# Quantitative CT Extent of Lung Damage in COVID-19 Pneumonia Is an Independent Risk Factor for Inpatient Mortality in a Population of Cancer Patients: A Prospective Study

**DOI:** 10.3389/fonc.2020.01560

**Published:** 2020-09-03

**Authors:** Toulsie Ramtohul, Luc Cabel, Xavier Paoletti, Laurent Chiche, Pauline Moreau, Aurélien Noret, Perrine Vuagnat, Pascal Cherel, Anne Tardivon, Paul Cottu, François-Clément Bidard, Vincent Servois

**Affiliations:** ^1^Department of Radiology, Institut Curie Paris & Saint Cloud, Paris, France; ^2^Department of Medical Oncology, Institut Curie Paris & Saint Cloud, Paris, France; ^3^INSERM U900 STAMPM Team, Institut Curie Paris & Saint Cloud, Paris, France; ^4^UVSQ, Université Paris-Saclay, Saint-Cloud, France

**Keywords:** COVID-19 pneumonia, quantitative chest CT extent, cancer patients, risk factor for mortality, prognostic performance

## Abstract

**Background:** CT lung extent has emerged as a potential risk factor of COVID-19 pneumonia severity with mainly semiquantitative assessment, and outcome was not assessed in the specific oncology setting. The main goal was to evaluate the prognostic role of quantitative assessment of the extent of lung damage for early mortality of patients with COVID-19 pneumonia in cancer patients.

**Methods:** We prospectively included consecutive cancer patients with recent onset of COVID-19 pneumonia assessed by chest CT between March 15, 2020, and April 20, 2020, and followed until May 1, 2020. Demographic, clinical, laboratory test data and imaging findings were recorded. Quantitative chest CT assessment of COVID-19 pneumonia was based on the density distribution of lung lesions using a freely available software recently released (Myrian XP-Lung). The association between extent of lung damage and overall survival was studied by univariate and multivariate Cox analysis. The Uno C-index was used to assess the discriminatory value of the quantitative CT extent of lung damage.

**Results:** Seventy cancer patients with chest CT evidence of COVID-19 were included. After a median follow-up of 25 days, 17 patients (24%) had died. The median quantitative chest CT extent of COVID-19 was 20% (IQR = 14–35, range = 3–59) for non-survivors vs. 10% (IQR = 6–15, range = 2–55) for survivors (*p* = 0.002). The extent of COVID-19 pneumonia was correlated with inpatient management (*p* = 0.003) and oxygen therapy requirements (*p* < 0.001). Independent factors associated with death were performance status (PS) ≥2 (HR = 3.9, 95% CI = [1.1–13.8] *p* = 0.04) and extent of COVID-19 pneumonia ≥30% (HR = 12.0, 95% CI = [2.2–64.4] *p* = 0.004). No differences were found regarding the histology of cancer, cancer stage, metastases sites, or type of oncologic treatment between the survivor and non-survivor groups. The cross-validated Uno C-index of the model including PS and extent of COVID-19 pneumonia was 0.83, 95% CI = [0.73–0.93].

**Conclusions:** The quantitative chest CT extent of COVID-19 pneumonia was a strong independent prognostic factor of early inpatient mortality in a population of cancer patients.

## Introduction

The outbreak of severe acute respiratory syndrome coronavirus 2 (SARS-CoV-2)-associated pneumonia (1), called coronavirus disease 2019 (COVID-19), started spreading across Europe in early March 2020, and, as of May 7, nearly 3,700,000 suspected cases and 260,000 deaths have been recorded worldwide, including 140,000 deaths in European countries (2). The COVID-19 pandemic has placed enormous pressures on health systems, and a variety of restriction policies have been adopted to reduce viral transmission. In the absence of effective treatment and herd immunity to this new coronavirus in the population (3), early diagnosis remains a key element to avoid spread of the disease, allowing quarantine and screening of patients with a history of exposure. The diagnosis of COVID-19 is based on reverse-transcription polymerase chain reaction (RT-PCR) on respiratory samples, including nasopharyngeal swabs, but the diagnostic performance of this test is limited by its only moderate sensitivity (63–72%) and its long turnaround times (4). Chest CT has rapidly emerged as a powerful tool for the diagnosis (5) of patients with COVID-19 in epidemic areas with higher sensitivity than RT-PCR (6). The causes of more severe forms of the disease are still under investigation but appear to be related to the lung damage induced by immune dysregulation in human SARS-CoV-2 infection (7). CT scores of the extent of lung damage based on visual semiquantitative assessment appeared to be associated with severity of symptoms, but these studies only focused on the radiological appearance without taking into account clinical and laboratory parameters, which make it difficult to draw any conclusions concerning patient management based on the extent of lung damage (8, 9). Chest CT assessment of the extent of COVID-19 lung damage has also not been performed in cancer patients, a population potentially at higher risk of severe infection due to a higher risk of immunodepression, comorbidities, and deterioration of the general condition (10). The primary objective of this study was to evaluate the prognostic role of the extent of COVID-19 pneumonia as assessed by quantitative chest CT in a population of cancer patients.

## Materials and Methods

### Study Population

This prospective study was conducted at the Institut Curie Hospitals (ICH) in Paris and St Cloud (France). The ICH is the largest cancer center in terms of the numbers of patients treated (11), and the Paris area registered more COVID-19 deaths than any other regions in France (12). All consecutive patients treated for cancer with chest CT evidence of COVID-19 pneumonia were prospectively included during the European COVID-19 epidemic outbreak between March 15, 2020, and April 20, 2020. Based on the COVID-19 reporting and data system, mandatory features for chest CT COVID-19 pneumonia were based on multifocal ground-glass opacities, with or without consolidations, reticular thickening, or subpleural bands (13). Chest CT examinations were requested for either clinical suspicion of COVID-19 pneumonia, history of exposure to confirmed COVID-19 cases, RT-PCR-positive swab, suspicion of pulmonary embolism, or routine cancer follow-up examination. Patients with no lung abnormalities were not included. Also, patients with preexisting equivocal findings before March 2020, such as GGO, were not included after comparison of the study chest CT scan with a previous CT scan (all imaging records for cancer patients treated at ICH are locally centralized). The following variables were prospectively recorded for each patient at chest CT examinations: age, sex, comorbidity, cancer type, tumor stage, sites of metastases, steroids or anticoagulant therapy, cancer treatment, date of onset of symptoms, type of symptoms, date and result of RT-PCR swab when performed, imaging features, and laboratory tests. The Eastern Cooperative Oncology Group performance status (PS) which determines the ability of patients to tolerate treatments in serious conditions was defined as follows: 0: asymptomatic; 1: symptomatic but completely ambulatory; 2: symptomatic, <50% in bed during the day; 3: symptomatic, >50% in bed, but not bedbound; 4: bedbound; and 5: dead. Cancer treatment was classified as chemotherapy, radiotherapy, immunotherapy, targeted therapy (including endocrine therapy), and surgery. Only treatment administered within 14 days of onset of symptoms were recorded. The protocol was approved by the participating center's local institutional review board and waived the informed consent due to the observational nature of the study. This report was written in accordance with STARD guidelines (14).

### Chest CT Image Interpretation

The onset of COVID-19 pneumonia at chest CT examinations served as baseline data. Chest CT scans were acquired with the patient in the supine position and with breath-holding following inspiration. Examinations were performed using a Somatom multi-slice CT scanner from Siemens Healthineers, Erlangen, Germany. A contrast agent was administered at the radiologist's discretion. The following technical parameters were used: detector collimation width: 0.625, tube current modulation: 100–250 mAs, and tube voltage: 120 kV. Image reconstruction parameters were as follows: slice thickness: 1–1.25 mm, iterative reconstruction Br40 (mediastinal) and BI57 (lung) kernels (Siemens Healthineers). Images were obtained with both mediastinal (width 400 HU; level 40 HU) and parenchymal (width 1500 HU; level −700 HU) window settings. All CT images were centrally transferred to a local PACS and analyzed by two radiologists blinded to baseline characteristics and outcome. Qualitative assessment was determined by consensus. In accordance with previous reports of COVID-19 imaging, the following features were recorded for each examination: ground-glass opacities (GGO), consolidation, septal thickening, cryptogenic organizing pneumonia (COP), air bronchogram, subpleural bands, peripheral/central topography, bilateral distribution, and upper/middle/lower lobe involvement (15). Non-COVID-19-related abnormalities were also evaluated: emphysema, lung or pleural metastases, nodes, and pleural effusion.

### Quantitative Assessment of the Extent of COVID-19 Pneumonia

**Figure 1:**
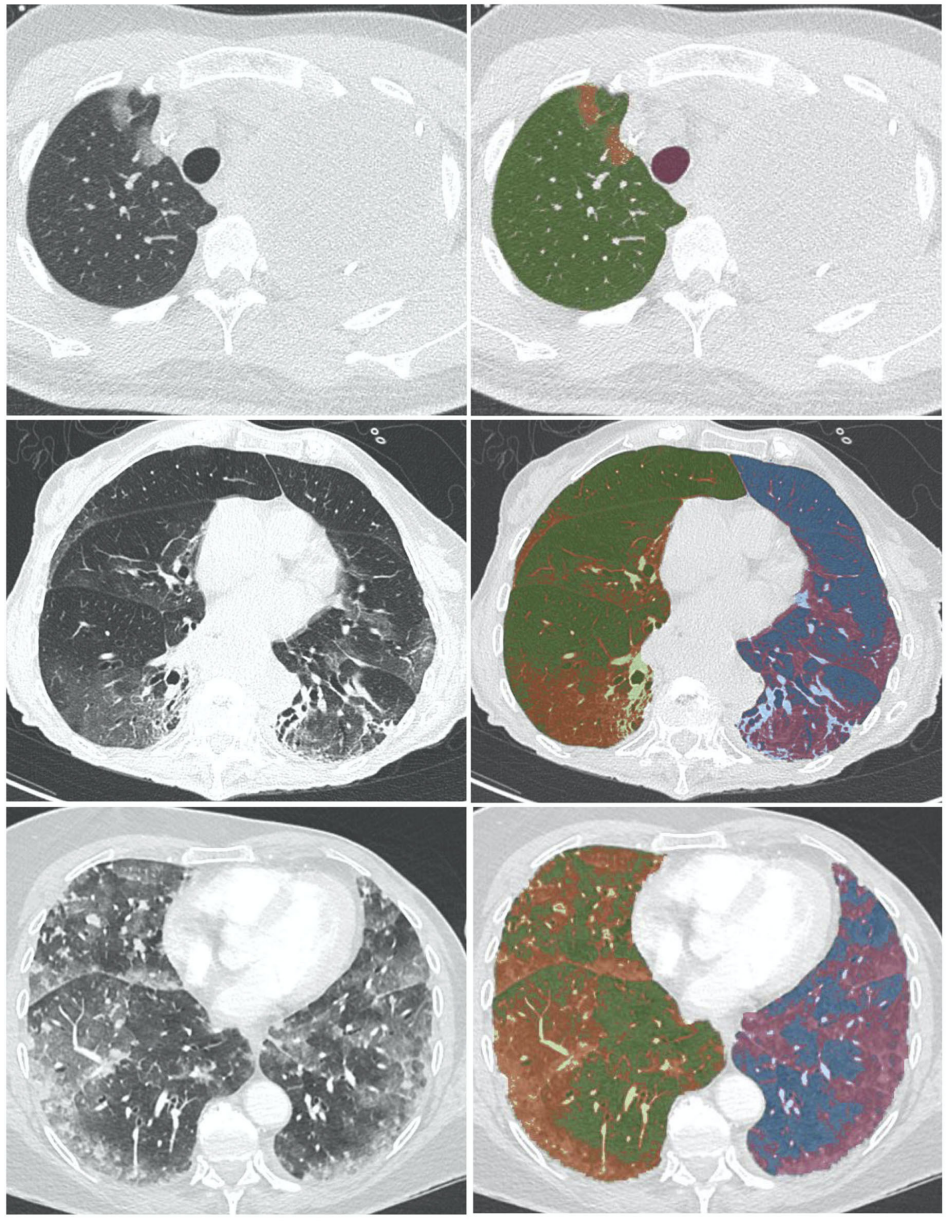
Examples of reconstructed images on lung window setting analyzed for quantitative assessment using Myrian XP-Lung software (version 1.19.1. Intrasense, Montpellier, France). Automatic segmentation of airways (pink), and left (green), and right (blue) lung parenchyma was performed. A slider was used with real-time colorization (red) to threshold COVID-19 opacities, such as GGO, and a second slider was used to threshold normal vascularization. From top to bottom, <14%, 14–30%, ≥30%.

Reconstructed images with lung window settings were then analyzed for quantitative assessment using Myrian XP-Lung software (version 1.19.1, Intrasense, Montpellier, France). Automatic segmentation of airways and left and right lung parenchyma was performed and manually edited, as necessary. The cumulative number of voxels was represented on a histogram of Hounsfield values allowing quantification of the density distribution and distinction between lung lesions and healthy lung tissue. A first slider was moved to threshold COVID-19 densities, such as GGO, and a second slider was set to threshold normal vascularization which may have a similar density to that of pneumonia, with real-time colorization. The following volumes were calculated in the histogram according to the thresholds: total lung volume and COVID-19 pneumonia volume, expressed in cm^3^. The extent of COVID-19 pneumonia was expressed as a percentage corresponding to the ratio of COVID-19 volume over total lung volume. Manual adjustment of segmentation was applied to disregard any non-COVID-19-related preexisting abnormalities (including pleural effusion, chest tumor, etc.) (Figure 1).

### Study Endpoints

The primary endpoint was overall survival, defined as the time from chest CT scan to death from any cause. Patients alive at the cutoff date on May 1, 2020 were censored at the last assessment date. Secondary outcomes were inpatient management and oxygen therapy requirements.

### Statistical Analysis

Continuous variables were analyzed by Mann–Whitney and Student tests according to distribution normality. Categorical variables were analyzed by χ^2^ test or Fisher's exact test. The Kaplan–Meier product-limit method was used to plot time to death, and comparisons between curves were performed with the exact log-rank test. The association between extent of lung damage and time to death was assessed by univariate and multivariate analyses using the Cox proportional hazard model. Schoenfeld residuals were used to check the proportional hazard assumption. For the multivariate analysis, we performed a backward selection approach, starting from the variables that were significant on univariate analysis at the 10% level (PS, SaO_2_, consolidation, pleural effusion, and chest CT extent of COVID-19 pneumonia). The Uno C-index, which is adapted to censored observation, was used to assess the discriminatory value of the CT extent of lung damage taken as a continuous variable; a value of 0.5 indicates no discriminatory ability, while value 1 would indicate perfect discrimination. Estimates overall and at various time points were calculated. To correct for over-optimism, 80 random splits for the 2-fold cross-validation was performed and the average C-index was provided. Confidence interval was obtained by bootstrap. In the absence of any prior cutoff value of the extent of COVID-19 lung damage in the oncologic setting, and as described by Gencer et al., the extent of lung damage was split in 3 ranges defined on the basis of two cutoff values: percentile 5 of the non-survivor group (with PS < 2) for the <14% range and percentile 95 of the survivor group for the ≥30% range (with PS < 2) (16). All analyses were performed with SAS software (version 9.4: SAS Institute, Cary, NC, USA) and R software. All statistical tests were two-sided, and a *p*-value less than 0.05 was considered statistically significant.

## Results

### Patient Characteristics

Between March 15 and April 20, 2020, 70 cancer patients with typical chest CT features of COVID-19 pneumonia were identified (Supplementary Figure 1). The clinical characteristics and laboratory results of the study population are summarized in Table 1. The median age of the population was 65 years (IQR = 52–72); 69% were female. Thirty-one patients (44%) presented at least one comorbid condition other than cancer. Twenty-seven patients (39%) had cardiovascular disease, most commonly hypertension (*n* = 22, 31%). Nine patients (13%) had diabetes mellitus, and 12 (17%) had a history of allergies. Nineteen patients (27%) were current or former smokers, and 5 patients (7%) had chronic obstructive pulmonary disease.

**Table 1:** Clinical characteristics and laboratory findings in cancer patients with COVID-19 pneumonia at chest CT examination.

**Variables**	**All** ***n* = 70**	**<14% extent** ***n* = 38**	**14–30% extent** ***n* = 23**	**≥30% extent** ***n* = 9**	***P*-value**
Age (years)	65 (52–72)	62.5 (45–69)	66 (55–76)	66 (52–79)	0.22
Sex					0.13
Male	22 (31%)	8 (21%)	10 (44%)	4 (44%)	
Female	48 (69%)	30 (79%)	13 (57%)	5 (56%)	
BMI (kg/m^2^)	24.1 (21–28)	24.6 (21–28)	22.5 (22–29)	23.2 (20–30)	0.82
Smoking					0.09
Current	9 (13%)	2 (5%)	4 (17%)	3 (33%)	
Former	10 (14%)	5 (13%)	5 (22%)	0 (0%)	
Non-smoker	51 (73%)	31 (82%)	14 (61%)	6 (67%)	
Comorbidities					
Cardiovascular disease	27 (39%)	16 (42%)	8 (35%)	3 (33%)	0.80
Hypertension	22 (31%)	12 (32%)	8 (35%)	2 (22%)	0.79
Diabetes type 2	9 (13%)	4 (11%)	4 (17%)	1 (11%)	0.73
COPD	5 (7%)	1 (3%)	3 (13%)	1 (11%)	0.27
Chronic renal failure	1 (1%)	1 (3%)	0 (0%)	0 (0%)	0.66
Hepatitis or cirrhosis	3 (4%)	1 (3%)	1 (4%)	1 (11%)	0.53
Autoimmune disease	3 (4%)	2 (5%)	1 (4%)	0 (0%)	0.78
Allergies	12 (17%)	5 (13%)	6 (26%)	1 (11%)	0.38
Time since onset of symptoms	6 (1–11)	7 (2–12)	5 (0–9)	6 (3–8)	0.41
Clinical examination					
Fever	37 (53%)	18 (47%)	14 (61%)	5 (56%)	0.58
Asthenia	6 (9%)	6 (16%)	0 (0%)	0 (0%)	0.06
Cough	26 (37%)	14 (37%)	11 (48%)	1 (11%)	0.16
Shortness of breath	33 (47%)	10 (26%)	14 (61%)	9 (100%)	** <0.001**
Ache	9 (13%)	5 (13%)	3 (13%)	1 (11%)	0.99
Anosmia or agueusia	6 (9%)	5 (13%)	1 (4%)	0 (0%)	0.30
Diarrhea	6 (9%)	4 (11%)	1 (4%)	1 (11%)	0.68
No symptoms	12 (17%)	8 (21%)	4 (17%)	0 (0%)	0.32
SaO_2_ ≤ 93%	25 (36%)	5 (13%)	12 (52%)	8 (89%)	** <0.001**
SARS-CoV-2 RT-PCR					0.34
Positive	44 (63%)	20 (53%)	18 (78%)	6 (67%)	
Negative	13 (18.5%)	9 (24%)	2 (9%)	2 (22%)	
Not done	13 (18.5%)	9 (23%)	3 (13%)	1 (11%)	
Hemoglobin (g/dL) (*n* = 66)^*^	10.8 (9–12)	10.9 (10–12)	11.3 (10–13)	10 (9–11)	0.28
White blood cells ( ×10^9^/L) (*n* = 67)^*^	5.4 (4–8)	4.5 (3–6)	6.8 (4–11)	6 (3–10)	**0.03**
Lymphocytes ( ×10^9^/L) (*n* = 68)^*^	0.7 (0.5–1.3)	0.7 (0.6–1.3)	0.7 (0.5–1.4)	0.7 (0.3–1)	0.77
Platelets ( ×10^9^/L) (*n* = 65)^*^	190 (126–256)	182 (122–236)	196 (128–263)	176 (145–283)	0.89
C-reactive protein (mg/L) (*n* = 56)^*^	73 (20–131)	29.5 (7–96)	102 (53–137)	157 (94–200)	**0.002**
Procalcitonin (ng/mL) (*n* = 41)^*^	0.1 (0.08–0.4)	0.1 (0.05–0.6)	0.1 (0.1–0.4)	0.8 (0.3–2)	0.21
Creatinine (mg/dL) (*n* = 68)^*^	0.7 (0.6–0.9)	0.7 (0.6–0.8)	0.7 (0.6–0.9)	0.6 (0.6–1.1)	0.77

*Data are expressed as median (IQR) or n (%). Ache refers to myalgia or headache. BMI, body mass index; COPD, chronic obstructive pulmonary disease; SaO_2_, oxygen saturation*.

* *Available data*.

*Bold p-values were considered statistically significant (p < 0.05)*.

The most common symptoms at onset of COVID-19 were fever (*n* = 37, 53%), shortness of breath (*n* = 33, 47%), and cough (*n* = 26, 37%). Subclinical forms were noted in 12 patients (17%), whereas 25 patients (36%) had SaO_2_ ≤ 93%. SARS-CoV-2 RT-PCR was positive in 44 patients (63%).

Median lymphocyte count was 0.7 ×10^9^/L (IQR = 0.5–1.3), and median C-reactive protein (CRP) was 73 mg/L (IQR = 20–131). Breast cancer was the most common cancer type, affecting 27 patients (39%), followed by lung (*n* = 11, 16%) and gastrointestinal cancer (*n* = 9, 13%). Forty-eight patients (69%) had stage IV metastatic disease, and 17 patients of them (35%) had lung metastases. Thirty-nine patients (56%) had received cancer treatment, most commonly chemotherapy (*n* = 27, 39%), during the 14 days preceding the onset of symptoms. Eighteen patients (26%) were treated with corticosteroids, and 8 patients (11%) were on anticoagulant therapy (Table 2).

**Table 2:** Tumor characteristics of patients with COVID-19 pneumonia.

**Variables**	**All** ***n* = 70**	**<14% extent** ***n* = 38**	**14–30% extent** ***n* = 23**	**≥30% extent** ***n* = 9**	***P*-value**
Cancer					0.83
Breast	27 (38%)	17 (45%)	7 (30%)	3 (34%)	
Lung	11 (16%)	6 (16%)	5 (22%)	0 (0%)	
Hemopathy	5 (7%)	2 (5%)	2 (9%)	1 (11%)	
Gynecologic	6 (9%)	4 (10%)	1 (4%)	1 (11%)	
GI	9 (13%)	3 (8%)	4 (17.5%)	2 (22%)	
Others	12 (17%)	6 (16%)	4 (17.5%)	2 (22%)	
PS					0.26
<2	43 (61%)	27 (71%)	12 (52%)	4 (44%)	
≥2	27 (39%)	11 (29%)	11 (48%)	5 (56%)	
Stage					0.99
Local	22 (31%)	12 (32%)	7 (30%)	3 (33%)	
Metastatic	48 (69%)	26 (68%)	16 (70%)	6 (67%)	
Metastatic site					
Lung	17/48 (35%)	9/26 (35%)	5/16 (31%)	3/6 (50%)	0.71
Brain	8/48 (17%)	4/26 (15%)	4/16 (25%)	0/6 (0%)	0.36
Liver	14/48 (29%)	5/26 (19%)	7/16 (44%)	2/6 (33%)	0.23
Bone	20/48 (42%)	10/26 (39%)	5/16 (31%)	4/6 (67%)	0.31
Peritoneum	5/48 (10%)	2/26 (8%)	1/16 (6%)	2/6 (33%)	0.14
Number of lines of treatment					0.23
<3	41/48 (85%)	24/26 (92%)	13/16 (81%)	4/6 (67%)	
≥3	7/48 (15%)	2/26 (8%)	3/16 (19%)	2/6 (33%)	
Oncologic treatment					
Surgery	1 (1%)	1 (3%)	0 (0%)	0 (0%)	0.65
Chemotherapy	27 (39%)	14 (37%)	10 (44%)	3 (33%)	0.74
Targeted or endocrine therapies	12 (17%)	10 (26%)	2 (9%)	0 (0%)	0.07
Immunotherapy	3 (4%)	2 (5%)	1 (4%)	0 (0%)	0.78
Radiotherapy	2 (3%)	1 (3%)	0 (0%)	1 (11%)	0.24
Regular treatment					
Corticosteroids	18 (26%)	9 (24%)	6 (26%)	3 (33%)	0.59
NSAID	3 (4%)	2 (5%)	1.0 (4%)	0 (0%)	0.52
Anticoagulants	8 (11%)	1 (3%)	6 (26%)	1 (11%)	**0.02**

*Data are expressed as n (%). Corticosteroids refer to a chronic daily dose equivalent to ≥10 mg of prednisolone (chemotherapy premedication was not taken into account); PS, performance status; NSAID, non-steroidal anti-inflammatory drugs*.

*Bold p-values were considered statistically significant (p < 0.05)*.

### Qualitative Chest CT Findings

The median duration of symptoms at the time of chest CT was 6 days (IQR = 1–11) for symptomatic cases. Chest CT scan with lung reconstruction was obtained on admission, and all patients showed features of COVID-19 pneumonia (Table 3). Ground-glass opacities with or without interlobular septal thickening and with or without consolidation were noted on all CT scans (*n* = 70, 100%). The other findings included consolidation (*n* = 10, 14%), COP (*n* = 8, 11%), and air bronchogram (*n* = 3, 4%). Fifty-three patients (76%) had bilateral lung involvement, and 63 patients showed predominant peripheral topography. Sixteen patients (23%) had pleural or lung metastases, 15 patients (21%) had pleural effusion, 5 patients (7%) had emphysema, and 3 patients (4%) had mediastinal nodes.

**Table 3:** CT findings in patients with COVID-19 pneumonia.

**Variables**	**All** ***n* = 70**	**<14% extent** ***n* = 38**	**14–30% extent** ***n* = 23**	**≥30% extent** ***n* = 9**	***P*-value**
COVID-19 features					
GGO	70 (100%)	38 (100%)	23 (100%)	9 (100%)	1.00
Consolidation	10 (14%)	5 (13%)	1 (4%)	4 (44%)	**0.02**
COP	8 (11%)	4 (11%)	3 (13%)	1 (11%)	0.96
Air bronchogram	3 (4%)	1 (3%)	1 (4%)	1 (11%)	0.53
Subpleural bands	18 (26%)	6 (16%)	10 (44%)	2 (22%)	0.06
Reticular thickening	10 (14%)	2 (5%)	6 (26%)	2 (22%)	0.06
Non-COVID-19-related abnormalities					
Emphysema	5 (7%)	2 (5%)	2 (9%)	1 (11%)	0.78
Metastases	16 (23%)	10 (26%)	6 (26%)	0 (0%)	0.22
Nodes	3 (4%)	1 (3%)	2 (9%)	0 (0%)	0.42
Pleural effusion	15 (21%)	7 (18%)	5 (22%)	3 (33%)	0.62
Distribution					**0.005**
Unilateral	17 (24%)	15 (40%)	2 (9%)	0 (0%)	
Bilateral	53 (76%)	23 (60%)	21 (91%)	9 (100%)	
Topography					0.48
Peripheral	63 (90%)	33 (87%)	21 (91%)	9 (100%)	
Central	7 (10%)	5 (13%)	2 (9%)	0 (0%)	
Lobes^*^					
Upper	54 (77%)	24 (63%)	21 (91%)	9 (100%)	**0.001**
Middle	37 (53%)	11 (29%)	17 (74%)	9 (100%)	** <0.001**
Lower	55 (79%)	26 (68%)	20 (87%)	9 (100%)	0.06
Lung volume (cm^3^)	3048 (2329–3937)	2839 (2046–3877)	3327 (2914–4138)	3293 (2425–3899)	0.34
Chest CT extent COVID-19 pneumonia (%)	11.8 (6–19)	6.6 (5–10)	17.8 (15–20)	37.5 (34–49)	** <0.001**

*Data are expressed as median (IQR) or n (%). GGO, ground glass opacities; COP, cryptogenic organizing pneumonia*.

* *Patients may present multiple lobe involvement*.

*Bold p-values were considered statistically significant (p < 0.05)*.

### Quantitative Chest CT Extent of COVID-19 Pneumonia

Quantitative chest CT extent of COVID-19 pneumonia was <14% in 38 of patients (54%), 14–30% in 23 patients (33%), and ≥30% in 9 patients (13%). Quantitative chest CT extent of COVID-19 pneumonia was significantly associated with shortness of breath, SaO_2_ ≤ 93%, white blood cells, C-reactive protein, anticoagulant therapy, consolidation, and bilateral distribution (*p* < 0.05) (Tables 1–3). Chest CT extent of COVID-19 lesions was correlated with inpatient management [respectively 19 (50%), 19 (83%), and 9 (100%) patients with <14%, 14–30%, or ≥30%, *p* = 0.003] and oxygen therapy requirements [respectively 8 (21%), 16 (70%), and 9 (100%) patients with <14%, 14–30%, or ≥30%, *p* < 0.001]. The time interval between symptom onset and chest CT for symptomatic cases (*n* = 57, 81%) was not correlated with quantitative chest CT extent of COVID-19 pneumonia with a median of 7 days (IQR = 2–12), 5 days (IQR = 0–9), and 6 days (IQR = 3–8) for <14%, 14–30%, or ≥30%, respectively, *p* = 0.41. Median lung volume was not significantly different according to the extent of lung damage on quantitative chest CT [2,839 cc (IQR = 2,046–3,877), 3,327 cc (IQR = 2,914–4,138), and 3,293 cc (IQR = 2,425–3,899) for <14%, 14–30%, or ≥30%, respectively, *p* = 0.34].

### Survival Outcome According to Quantitative CT Extent of COVID-19 Pneumonia

**Figure 2:**
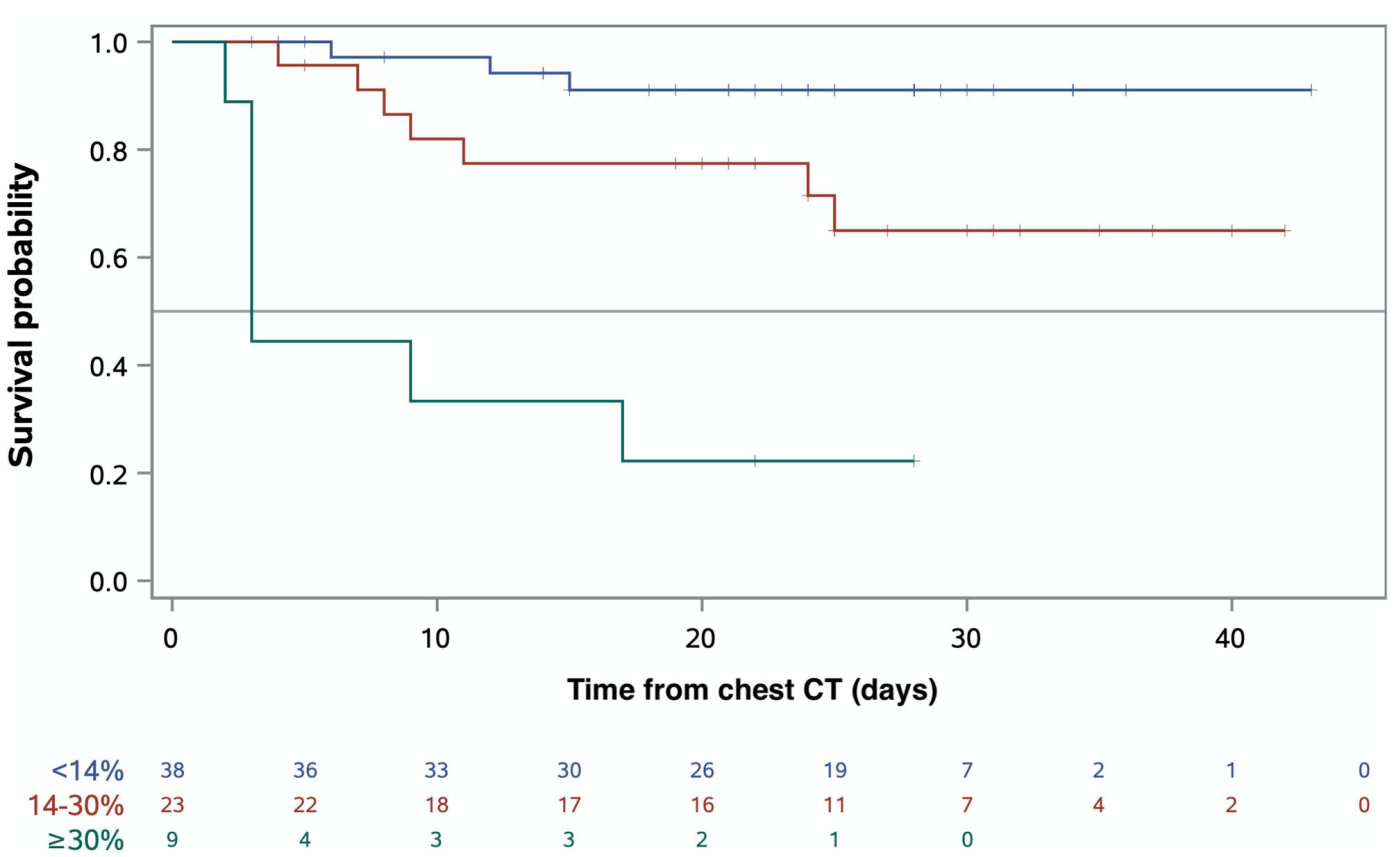
Prognostic impact of CT extent of COVID-19 pneumonia. Kaplan–Meier survival estimates among patients with extent of lung damage <14%, 14–30%, and ≥30%.

**Figure 3:**
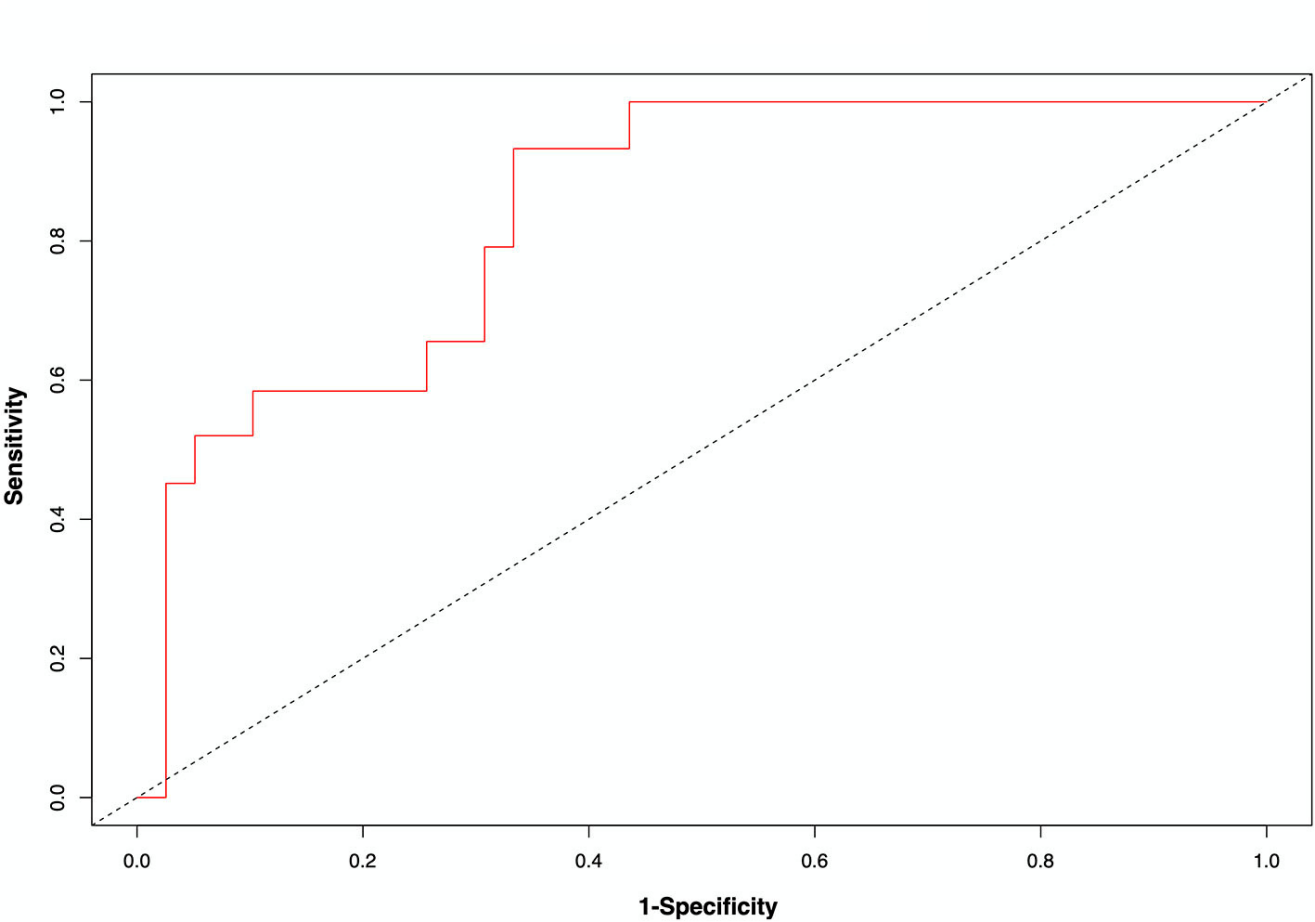
Receiver operating characteristic curve for the model including performance status and chest CT extent of COVID-19 pneumonia at day 21 (Uno statistic 0.83, 95% CI = [0.73–0.93]).

Median follow-up was 25 days (95% CI = [23–28]). At the cutoff date, 17 patients (24%) had died. The median quantitative chest CT extent of COVID-19 pneumonia was 20% (IQR = 14–35, range: 3–59) for non-survivors vs. 10% (IQR = 6–16, range: 2–55) for survivors (*p* = 0.002). The extent of lung damage on quantitative chest CT was associated with death with 7 (78%), 8 (30%), and 3 (8%) deaths for an extent of damage ≥30%, 14–30%, and <14%, respectively (*p* < 0.001). Figure 2 depicts the Kaplan–Meier survival estimates according to quantitative chest CT extent of COVID-19 pneumonia. Overall survival (OS) decreased significantly with increasing extent of lung damage (*p* < 0.001). For patients with COVID-19 pneumonia involving ≥30% of the lungs, median survival was 3 days. On univariate analysis (Supplementary Table 1), variables associated with death were PS ≥ 2 (HR = 4.8, 95% CI = [1.7–13.6], *p* = 0.003), shortness of breath (HR = 10.2, 95% CI = [2.3–44.8], *p* = 0.002), SaO_2_ ≤ 93% (HR = 6.1, 95% CI = [2.0–18.6], *p* = 0.002), consolidation (HR = 3.1, 95% CI = [1.1–9.0], *p* = 0.03), pleural effusion (HR = 3.7, 95% CI = [1.4–9.6], *p* = 0.007), chest CT extent of COVID-19 lesions of 14–30% (HR = 3.9, 95% CI = [1.0–15.2], *p* = 0.047), and chest CT extent of COVID-19 lesions ≥30%, (HR = 18.8, 95% CI = [4.8–73.8], *p* < 0.001). On multivariate analysis (Table 4), the only two independent factors associated with death were PS ≥ 2 (HR = 3.9, 95% CI = [1.1–13.8], *p* = 0.04) and extent of COVID-19 lesions ≥30% (HR = 12.0, 95% CI = [2.2–64.4], *p* = 0.004). Figure 3 illustrates the area under the receiver operating characteristics curve, at day 21, for the model including PS and chest CT extent of COVID-19 pneumonia. The <14% cutoff had a sensitivity of 79.3% and a specificity of 59.0% to predict mortality, while the ≥30% cutoff had a sensitivity of 42.0% and a specificity of 94.9% (assessed at day 21). The model including PS and chest CT extent of COVID-19 pneumonia showed a higher prognostic performance after correction for over-optimism (Uno statistic 0.83, 95% CI = [0.73–0.93]) as compared to the clinical model with PS alone (Uno statistic 0.70, 95% CI = [0.56–0.83]) or model with chest CT extent of COVID-19 only (Uno statistic 0.75, 95% CI = [0.61–0.89]).

**Table 4:** Univariate and multivariate analysis of death.

**Variables**		**Univariate analysis**		**Multivariate analysis**	
		**HR (95% CI)**	***P***	**HR (95% CI)**	***p***
Age (years)	<65 (vs. ≥65)	2.1 (0.8–5.6)	0.16		
Sex	Male (vs. female)	1.5 (0.6–4.0)	0.39		
BMI (kg/m^2^)	<30 (vs. ≥30)	3.7 (0.5–27.9)	0.20		
Cancer stage	Metastatic (vs. non-metastatic)	1.2 (0.4–3.4)	0.74		
PS	≥2 (vs. <2)	4.8 (1.7–13.6)	**0.003**	3.9 (1.1–13.8)	**0.04**
Treatment	Steroids (vs. none)	2.2 (0.8–5.7)	0.12		
	Anticoagulants (vs. none)	0.5 (0.1–3.6)	0.47		
Time from symptoms onset (days)	≥6 (vs. <6)	0.8 (0.3–2.1)	0.58		
Shortness of breath	Yes (vs. no)	10.2 (2.3–44.8)	**0.002**		
SaO_2_	≤ 93% (vs. >93%)	6.1 (2.0–18.6)	**0.002**	1.5 (0.4–6.2)	0.58
Lymphocytes	<1 ×10^9^/L (vs. ≥1 ×10^9^/L)	1.1 (0.4–2.9)	0.92		
Consolidation	Yes (vs. no)	3.1 (1.1–9.0)	**0.03**	3.0 (0.9–10.2)	0.07
Bilateral distribution	Yes (vs. no)	1.0 (0.3–3.2)	0.95		
Pleural effusion	Yes (vs. no)	3.7 (1.4–9.6)	**0.007**	1.5 (0.5–4.7)	0.54
SARS-CoV-2 RT-PCR			0.25		
	Positive (vs. negative)	2.1 (0.5–9.0)	0.34		
	Not done (vs. negative)	0.5 (0.04–5.0)	0.52		
C-reactive protein (mg/L)	≥89 (vs. <89)	2.2 (0.8–6.0)	0.15		
Chest CT extent of COVID-19 pneumonia			** <0.001**		**0.01**
	14–30% (vs. <14%)	3.9 (1.0–15.2)	**0.047**	3.4 (0.7–16.9)	0.14
	≥30% (vs. <14%)	18.8 (4.8–73.8)	** <0.001**	12.0 (2.2–64.4)	**0.004**

*Shortness of breath was excluded from the univariate analysis because of collinearity with SaO_2_. HR, hazard ratio; CI, confidence interval; BMI, body mass index; PS, performance status; SaO_2_, oxygen saturation*.

*Bold p-values were considered statistically significant (p < 0.05)*.

## Discussion

This prospective study conducted in a large cancer center assessed the extent of COVID-19 pneumonia on quantitative chest CT as a prognostic factor for early in-hospital mortality in cancer patients. A strong point of this study conducted in a cancer center is the availability of imaging history, allowing the distinction between COVID-19 pneumonia and non-specific CT findings. Some authors have raised concerns about the specificity of chest CT to distinguish COVID-19 pneumonia from other infectious or neoplastic diseases associated with similar CT findings (17). It should be noted that the positive predictive value of CT will be higher in the context of high prevalence of COVID-19 infection during the study period (18). Moreover, in our study, RT-PCR confirmed the suspicion of COVID-19 observed on chest CT in 63% of cases, similar to the findings reported in a previous radiological study (6).

To the best of our knowledge, this is the first report of the extent of lung damage, as assessed by quantitative chest CT, as an independent prognostic factor for early in-hospital mortality in patients with COVID-19, with the advantage of freely available software. With automatic segmentation and nearly instantaneous results based on distribution of opacities, quantitative CT assessment may be a more reliable method to evaluate the extent of pneumonia than a semiquantitative score based on the radiologist's visual assessment (9, 19, 20). Automated tools, such as deep learning-based quantitative CT, are also being evaluated and could represent time-saving solutions (21, 22). As reported by Li and Li et al., the prognostic role of CT lung scores was limited by important unbalanced characteristics between groups, including age and comorbidities, and most of the evaluated cases were still hospitalized at the time of analysis (8, 23). Imaging risk factors for inpatient mortality therefore could not be assessed. In our study, the extent of COVID-19 pneumonia was twofold higher in the non-survivor group (20 vs. 10%) and the survival probability decreased with increasing extent of lung damage. We also observed a clear correlation between the extent of COVID-19 pneumonia and oxygen therapy requirements. Our proposed 30% cutoff for extent of disease, associated with a high specificity rate to predict mortality, was much lower than the “severe” (50–75%) or “critical” threshold (>75%) defined by the French Radiology Society to stratify pneumonia severity. With quantification of well-aerated lung parenchyma (WAL), Colombi et al. determined that WAL <73% was correlated with ICU admission or death (24). This highlights the need for a specific score for cancer patients, including other variables such as PS, as this population may have a poorer prognosis (25). The models including PS and additional chest CT evaluation of COVID-19 pneumonia demonstrated the highest prognostic performance (Uno statistic 0.83, 95% CI = [0.73–0.93]) after cross-validation. By taking account of quantitative chest CT extent of COVID-19 pneumonia, we did not find any significant differences in terms of survival according to tumor histology, cancer stage, metastatic sites, or type of cancer treatment.

No correlation was observed between time from onset of symptoms and quantitative chest CT extent of COVID-19 pneumonia; however, a large majority of patients were symptomatic at chest CT examinations. Several studies have reported that lung inflammation is the main cause of life-threatening disorders in severe COVID-19 and that severity may be related to an inappropriate reaction of the immune system resulting in cytokine storm (26–28). Quantitative assessment of the extent of COVID-19 pneumonia, performed at a symptomatic stage (median time from symptoms onset of 6 days), may reflect the immune rebound effect that can complicate the management of patients with a reassuring initial presentation with no other known risk factors. Concerning qualitative imaging features, consolidation was found to be correlated with poorer outcomes (29, 30). In our study, consolidation was no longer significant in Cox analysis when chest CT extent of the COVID-19 lesions was considered. A longitudinal study of temporal changes demonstrated that the proportion of pure GGO decreased whereas the proportion of higher density abnormalities increased within the first 3 weeks of symptoms onset (31).

This study has several limitations. It only reported the early inpatient mortality due to the short follow-up, and the sample may not be representative of all cancer patients in terms of clinical setting and tumor type (32). An external validation cohort of this quantitative chest CT score must therefore be conducted to confirm these results.

In this prospective study of patients undergoing CT imaging of COVID-19 pneumonia, we found that the quantitative chest CT extent of COVID-19 pneumonia was a strong independent prognostic factor of early in-hospital mortality in a population of cancer patients. Quantitative chest CT should be proposed to a greater number of cancer patients in order to identify patients at risk and to tailor patients' management.

## Data Availability Statement

The raw data supporting the conclusions of this article will be made available by the authors, without undue reservation.

## Ethics Statement

The studies involving human participants were reviewed and approved by Institut Curie institutional review board. Written informed consent for participation was not required for this study in accordance with the national legislation and the institutional requirements.

## Author Contributions

TR and VS: guarantors of integrity of the entire study and literature research. TR, VS, XP, LCa, and F-CB: study concepts and study design or data acquisition or data analysis and interpretation. TR and XP: statistical analysis. All authors: manuscript editing and manuscript final version approval.

## Conflict of Interest

The authors declare that the research was conducted in the absence of any commercial or financial relationships that could be construed as a potential conflict of interest.

## Abbreviations

CT: computed tomography
RT-PCR: reverse transcription-polymerase chain reaction
GGO: ground glass opacities
COP: cryptogenic organizing pneumonia
PS: performance status
CRP: C-reactive protein
HR: hazard ratio
95% CI: 95% confidence interval.
