# Supplementary material for: Quantitative CT Extent of Lung Damage in COVID-19 Pneumonia Is an Independent Risk Factor for Inpatient Mortality in a Population of Cancer Patients: A Prospective Study
Source: Front Oncol. 2020 Sep 3;10:1560. doi: 10.3389/fonc.2020.01560 (PMC7494966; doi:10.3389/fonc.2020.01560)
Supplement: Supplementary file 1 [file Data_Sheet_1.docx]

**Supplementary Material**

**Supplementary Figure 1.** Flow chart

CT, computerized tomodensitometry; RT-PCR, reverse transcriptase – polymerase chain reaction.**Supplementary Table 1.** Univariate and multivariate analysis of death

| **Variables** | | **Univariate analysis** | | **Multivariate analysis** | |
| --- | --- | --- | --- | --- | --- |
|  |  | **HR (95% CI)** | **p** | **HR (95% CI)** | **p** |
| Comorbidites | Cardiovascular (vs no cardiovascular) | 1.5 (0.6-4.0) | 0.39 |  |  |
|  | Hypertension (vs no hypertension) | 1.2 (0.5-3.3) | 0.70 |  |  |
|  | Diabetes (vs no diabetes) | 1.0 (0.2-4.2) | 0.94 |  |  |
|  | COPD (vs no COPD) | 0.8 (0.1-5.6) | 0.78 |  |  |
|  | Allergies (vs no allergies) | 1.1 (0.3-3.4) | 0.97 |  |  |
| Fever | Yes (vs no) | 0.7 (0.3-1.8) | 0.43 |  |  |
| Systolic blood pressure (mmHg) | ≤90 (vs >90) | 5.5 (0.7-43.4) | 0.11 |  |  |
| Diastolic blood pressure (mmHg) | ≤50 (vs >50) | 2.1 (0.3-16.0) | 0.48 |  |  |
| Heart rate (bpm) | ≥100 (vs <100) | 1.4 (0.5-3.7) | 0.56 |  |  |
| White blood cells (x 10^9^/L) | ≥10 (vs <10) | 1.9 (0.6-5.9) | 0.26 |  |  |
| Hemoglobin (g/dL) | ≤10 (vs >10) | 2.3 (0.9-5.9) | 0.09 |  |  |
| Creatinine (µmol/L) | ≥65 (vs <65) | 1.8 (0.7-4.7) | 0.22 |  |  |
| Procalcitonin (ng/mL) | >0.1 (vs <0.1) | 3.3 (0.7-15.3) | 0.12 |  |  |
| Cancer | Breast (vs no breast) | 0.7 (0.2-2.0) | 0.48 |  |  |
| Metastatic sites | Lung (vs no lung) | 1.4 (0.5-4.0) | 0.52 |  |  |
| Number of lines of treatment | ≥3 (vs <3) | 2.2 (0.6-8.1) | 0.25 |  |  |
| Cancer treatments | Chemotherapy (vs no chemotherapy) | 0.8 (0.3-2.1) | 0.59 |  |  |
|  | Radiotherapy (vs no radiotherapy) | 3.2 (0.4-24.7) | 0.26 |  |  |
| Regular treatments | NSAID (vs no NSAID) | 1.2 (0.2-9.1) | 0.86 |  |  |
| BOOP | Yes (vs no) | 0.4 (0.06-3.3) | 0.43 |  |  |
| Reticular thickening | Yes (vs no) | 1.3 (0.4-4.7) | 0.65 |  |  |
| Air bronchogram | Yes (vs no) | 1.8 (0.2-13.3) | 0.58 |  |  |
| Subpleural bands | Yes (vs no) | 1.5 (0.6-4.1) | 0.42 |  |  |
| Emphysema | Yes (vs no) | 1.7 (0.4-7.6) | 0.46 |  |  |
| Topography | Central (vs peripheral) | 2.2 (0.6-7.6) | 0.22 |  |  |

HR, Hazard Ratio; CI, Confidence Interval; NSAID. Non-steroidal anti-inflammatory drugs; Ground Glass Opacities; COP, cryptogenic organizing pneumonia.
